# Effect of antenatal milk expression on prenatal breastfeeding self-efficacy, lactogenesis onset, and early exclusive breastfeeding in pregnant women with gestational diabetes mellitus: a randomized controlled trial

**DOI:** 10.1186/s13006-026-00879-w

**Published:** 2026-08-06

**Authors:** Fengcheng Cai, Zhangyue Jia, Yingying Wu, Mengyu Zeng, Zhenyi Wang, Jingke Yang, Mengyan Xu, Yina Zhang

**Affiliations:** 1 Department of Nursing, Hangzhou Women’s Hospital (Hangzhou Maternity and Child Health Care Hospital), Hangzhou, Zhejiang 310008 China; 2 School of Nursing, Zhejiang Chinese Medical University, Hangzhou, Zhejiang 310008 China; 3 Department of Nursing, Women’s Hospital School of Medicine Zhejiang University, Hangzhou, Zhejiang 310006 China

**Keywords:** Gestational diabetes mellitus, Antenatal milk expression, Breastfeeding self-efficacy, Lactogenesis onset, Exclusive breastfeeding

## Abstract

**Background:**

Pregnant women with gestational diabetes mellitus (GDM) frequently experience delayed onset of lactogenesis, insufficient breastfeeding self-efficacy, and low rates of early exclusive breastfeeding, highlighting the need for effective interventions.

**Objective:**

To investigate the effects of antenatal milk expression (AME) on prenatal breastfeeding self-efficacy, lactogenesis onset, and early exclusive breastfeeding in pregnant women with GDM.

**Methods:**

In this single-center randomized controlled trial conducted at Hangzhou Women’s Hospital (Hangzhou Maternity and Child Health Care Hospital) in China, 240 women with GDM in late pregnancy were enrolled and randomly assigned in a 1:1 ratio to an intervention group or a control group. The control group received routine prenatal care, whereas the intervention group received routine care plus AME. Prenatal breastfeeding self-efficacy, time to lactogenesis onset, incidence of delayed onset of lactogenesis II, exclusive breastfeeding rates at 24 h postpartum and before hospital discharge, as well as social support and breastfeeding behavior prediction indicators, were compared between the two groups.

**Results:**

A total of 233 participants completed the study. Compared with the control group, the intervention group showed significantly higher total and domain scores for prenatal breastfeeding self-efficacy; a significantly shorter time to lactogenesis onset [(41.15 ± 21.94) h vs. (55.74 ± 23.29) h]; a significantly lower incidence of delayed onset of lactogenesis II (8.6% vs. 24.8%); and significantly higher exclusive breastfeeding rates at 24 h postpartum and before discharge (81.9% vs. 66.7%, 43.1% vs. 28.2%). In addition, improvements in social support and breastfeeding behavior prediction indicators were more pronounced in the intervention group. No statistically significant differences in maternal or neonatal outcomes were observed between the groups, and no AME-related maternal or neonatal adverse events were identified during safety monitoring throughout the study period.

**Conclusion:**

AME improved prenatal breastfeeding self-efficacy in women with GDM, was associated with earlier lactogenesis onset and a lower incidence of delayed onset of lactogenesis II, and increased early in-hospital exclusive breastfeeding rates. These breastfeeding findings should be interpreted as early postpartum outcomes rather than evidence of sustained exclusive breastfeeding beyond discharge. When implemented with standardized instruction and safety monitoring in eligible women with GDM, AME may serve as a useful antenatal component of perinatal breastfeeding support.

**Supplementary Information:**

The online version contains supplementary material available at https://doi.org/10.1186/s13006-026-00879-w.

## Introduction

Gestational diabetes mellitus (GDM) refers to glucose intolerance first identified or diagnosed during pregnancy and is one of the most common medical complications of pregnancy [1, 2]. Its core pathophysiological basis is aggravated insulin resistance resulting from inadequate compensatory pancreatic β-cell function, and it shares certain mechanistic links with type 2 diabetes mellitus [1, 2]. GDM not only increases the risk of adverse perinatal outcomes, including gestational hypertension, preeclampsia, cesarean delivery, and neonatal hypoglycemia, but also raises the long-term risk of diabetes and cardiovascular disease in both mothers and their offspring [3, 4]. The prevalence of GDM continues to rise worldwide, affecting approximately 14% to 16.5% of pregnancies globally and reaching as high as 23% in some developed regions, making it a major public health concern [5, 6]. The burden of GDM is also substantial in China and other low- and middle-income settings. A recent systematic review and meta-analysis reported an increasing prevalence of GDM in China over the past three decades, with a pooled prevalence of 15.6% under the International Association of Diabetes and Pregnancy Study Groups criteria after 2010 [7]. Globally standardized estimates further indicate high regional burdens in South-East Asia and the Middle East and North Africa, underscoring the need for scalable perinatal interventions across diverse healthcare settings [8]. With the increasing proportion of advanced maternal age pregnancies, the growing prevalence of obesity, and the influence of socioeconomic factors, GDM has become a substantial public health challenge and places a considerable burden on healthcare systems [5, 9]. In addition, women with GDM are more likely to experience delayed lactogenesis onset, lower exclusive breastfeeding rates, and earlier introduction of formula feeding [10, 11], which not only compromises neonatal nutrition and metabolic homeostasis but also undermines maternal confidence in breastfeeding and its continuation [11, 12]. Existing interventions have mainly focused on postpartum education, specialist nursing care, and social support; however, these approaches are often implemented after delivery and may provide insufficient antenatal skill preparation and confidence-building for women with GDM. Moreover, current evidence remains limited regarding their effectiveness in simultaneously reducing the risk of delayed lactogenesis and improving breastfeeding self-efficacy [12–15].

As a lactation preparation strategy initiated in late pregnancy, antenatal milk expression (AME) has gradually been introduced into the care of high-risk pregnant women in recent years. Previous studies have suggested that AME is safe and feasible, may facilitate the onset of milk secretion, improve breastfeeding self-efficacy and maternal satisfaction, and may also help increase exclusive breastfeeding rates, shorten the time to lactogenesis onset, and improve maternal and neonatal outcomes in women with GDM [16, 17]. The Diabetes and Antenatal Milk Expressing randomized controlled trial supported the safety of AME in carefully selected low-risk women with diabetes in pregnancy [18]. However, evidence remains limited regarding whether AME improves the integrated antenatal-to-early-postpartum breastfeeding pathway, particularly prenatal breastfeeding self-efficacy, lactogenesis onset, and early exclusive breastfeeding among women with GDM in mainland China. Therefore, this study focused on the effects of AME on prenatal breastfeeding self-efficacy, lactogenesis onset, and early exclusive breastfeeding in women with GDM, with the aim of improving antenatal lactation support systems, increasing breastfeeding rates in this special population, and providing evidence-based support and an innovative approach for breastfeeding management strategies in women with GDM.

## Methods

### Study design and participants

This was a single-center randomized controlled trial. Using a consecutive sampling approach, pregnant women with gestational diabetes mellitus (GDM) in late pregnancy who attended the obstetric outpatient clinic of a tertiary Grade A specialty hospital in Zhejiang Province between March and July 2024 were recruited if they met the inclusion and exclusion criteria, voluntarily agreed to participate, and provided written informed consent. The study followed a prespecified protocol approved by the Ethics Committee of Hangzhou Women’s Hospital and was reported in accordance with the CONSORT statement. Key protocol elements, including eligibility criteria, sample size estimation, randomization, blinding, interventions, outcome measures, safety monitoring, and statistical analysis, are described in the Methods section. The completed CONSORT checklist is provided as Supplementary File 1. The trial was registered in the Chinese Clinical Trial Registry (registration number: ChiCTR2500109811). Throughout the study, participant privacy and data security were strictly protected. If any discomfort or abnormal condition occurred during the intervention period, the intervention was discontinued immediately and appropriate assessment and management were provided.

### Eligibility criteria and diagnostic criteria for GDM

The diagnosis of GDM was based on the criteria of the International Association of Diabetes and Pregnancy Study Groups (IADPSG) [19] A 75-g oral glucose tolerance test (OGTT) was performed at 24–28 weeks of gestation. GDM was diagnosed when any one of the following thresholds was met: fasting plasma glucose 5.1–6.9 mmol/L, 1-h plasma glucose ≥ 10.0 mmol/L, or 2-h plasma glucose 8.5–11.0 mmol/L. Only women with GDM managed primarily through dietary and lifestyle intervention were included. Women requiring insulin or oral hypoglycemic therapy were not included to reduce clinical heterogeneity related to glycemic severity, obstetric risk, neonatal risk, and breastfeeding-related outcomes.

The inclusion criteria were as follows: (1) fulfillment of the diagnostic criteria for gestational diabetes mellitus (GDM); (2) age ≥ 18 years and < 35 years; (3) gestational age ≥ 36 weeks; (4) singleton pregnancy; (5) primiparity; and (6) a clear intention to breastfeed.

The exclusion criteria were as follows: (1) the presence of other pregnancy-related complications or medical/surgical comorbidities, such as gestational hypertension, hypothyroidism complicating pregnancy, placenta previa, or antepartum hemorrhage; (2) threatened preterm labor; (3) fetal growth restriction; (4) non-reassuring fetal heart rate monitoring; (5) unsuitability for breastfeeding due to maternal illness, medication use, or other related factors; (6) flat or inverted nipples; (7) conception by in vitro fertilization and embryo transfer; (8) psychological or psychiatric disorders; and (9) fetal malformations. Flat or inverted nipples were assessed by a trained obstetric nurse during routine prenatal breast and nipple examination before randomization. This assessment was completed before allocation and was independent of subsequent outcome assessment. Women with flat or inverted nipples were excluded because nipple anatomy may affect antenatal hand expression, early latch, and breastfeeding establishment.

The withdrawal criteria were: (1) prior participation in other similar breastfeeding intervention studies identified before study initiation, which might affect the evaluation of the present intervention; and (2) erroneous enrollment of participants who did not meet the inclusion criteria.

The dropout criteria were: (1) delivery outside the study hospital; (2) failure to complete the full intervention protocol; and (3) voluntary withdrawal from the study during the the study period.

### Sample size estimation, randomization, and blinding

The sample size was calculated using the formula for comparing two independent proportions. The exclusive breastfeeding rate before hospital discharge was used as the main parameter for sample size estimation. Based on previous studies of breastfeeding outcomes in women with GDM and the expected effect of AME, a two-sided significance level of α = 0.05 and a statistical power of 1 − β = 0.90 were used. Accordingly, the required sample size was estimated to be 96 participants per group. Allowing for an anticipated attrition rate of approximately 20%, the final target sample size was set at 240 participants, who were allocated in a 1:1 ratio to the intervention group and the control group, with 120 participants in each group.

Randomization was performed using a random number table. An independent researcher who was not involved in participant recruitment, intervention delivery, outcome assessment, or data analysis generated the random allocation sequence. Allocation concealment was ensured using sequentially numbered, sealed, opaque envelopes. Participants were assigned study numbers consecutively according to the order of enrollment, and group allocation was determined by sequentially opening the envelopes. Because the AME intervention required face-to-face implementation, neither the participants nor the intervention providers were blinded. However, blinding was maintained for data collectors and statistical analysts to minimize measurement and analytical bias.

### Interventions

#### Control group

Participants in the control group received routine prenatal care for GDM, including standard antenatal care, dietary and exercise management, guidance on blood glucose monitoring, and breastfeeding-related health education. Routine care was provided according to national and institutional guidance for GDM management and perinatal care. After delivery, participants received routine postpartum nursing care, including maternal and neonatal observation, ward-based breastfeeding guidance, neonatal feeding assessment when clinically indicated, discharge education, and routine postpartum follow-up arranged by the hospital. No structured antenatal AME training, AME skill demonstration, or AME-related home practice supervision was provided in the control group.

#### Intervention group

In addition to routine care, participants in the intervention group received an antenatal milk expression (AME) intervention. Before participant recruitment for the present randomized trial, the AME intervention protocol was developed through a literature review, qualitative interviews with women with GDM and obstetric healthcare professionals, a clinical pilot study, and research team discussions. These preparatory procedures were completed before the present RCT and did not involve participants enrolled in the trial. The protocol was guided by the extended Theory of Planned Behavior.

After 36 weeks of gestation, participants received systematic guidance addressing behavioral attitudes, subjective norms, perceived behavioral control, behavioral intention, and implementation intention, together with support from spouses, peers, and healthcare professionals. AME training was administered by uniformly trained obstetric nurses with lactation counseling experience. Training was conducted in the obstetric outpatient education room using one-to-one instruction, including health education, skill demonstration, supervised practice, and return demonstration. Each initial training session lasted approximately 30–40 min. Participants began home practice only after completing the in-clinic skill assessment. From 37 weeks of gestation onward, AME skills training was introduced, consisting primarily of breast massage and hand expression. During breast massage, the areola and nipple were avoided, and each breast was massaged for 3–5 min per session. For hand expression, participants assumed a comfortable forward-leaning seated or upright position. The thumb and index finger were placed in a “C” shape approximately 2.5 cm from the base of the nipple, and gentle backward pressure toward the chest wall followed by compression was applied. The two breasts were expressed alternately, with hand expression performed for 5 min on each side. The intervention was administered twice daily, once in the morning after waking and resting and once before bedtime, and continued until delivery. Participants were instructed to avoid AME immediately after walking or other physical activity and to postpone practice in the presence of fatigue, abdominal tightening, or suspected uterine contractions.

Spouses and primary family caregivers, including mothers or mothers-in-law when they were expected to provide postpartum care, were invited to participate in AME-related education. Family involvement included emotional support, practice reminders, assistance with milk storage, and support for breastfeeding-related decisions. Peer support was facilitated through participant communication under the guidance of the research team, while medical questions were addressed by healthcare professionals. If participants perceived uterine contractions during the procedure, they were instructed to stop immediately and resume only after the contractions had subsided, depending on their condition. During home practice, participants recorded each AME session, milk expression status, fetal movement, uterine contractions, vaginal bleeding, and other discomforts in a daily practice log. The log was reviewed daily by the research team through online communication. Participants were instructed to stop AME immediately and seek obstetric assessment if regular or persistent uterine contractions, decreased fetal movement, vaginal bleeding, leakage of fluid, persistent abdominal pain, or other abnormal symptoms occurred. After passing the in-clinic AME skills assessment and completing six consecutive practice sessions, participants in the intervention group underwent repeat assessment with the relevant questionnaires. If milk was expressed during the procedure, all milk collected within 24 h was stored in clean breast milk storage bags and frozen for postpartum use.

### Outcome measures

#### Baseline data

At baseline, general demographic and pregnancy-related data were collected, including age, pre-pregnancy body mass index (BMI), gestational weight gain, ethnicity, marital status, educational level, occupation, annual per-capita household income, planned feeding method during hospitalization, payment source, and maternity leave duration.

#### Primary outcomes

1. Prenatal breastfeeding self-efficacy: Prenatal breastfeeding self-efficacy was assessed using the Prenatal Breastfeeding Self-Efficacy Scale (PBSES) [20]. This instrument measures pregnant women’s confidence in and perceived ability to successfully initiate and maintain breastfeeding after delivery. The PBSES consists of 20 items scored on a 5-point Likert scale, with each item scored from 1 to 5. The total score ranges from 20 to 100, with higher scores indicating higher prenatal breastfeeding self-efficacy. In the present study, the total score and four domain scores, including behavioral perseverance, skills and needs, information acquisition, and embarrassment, were calculated according to the original scoring method. Assessments were performed at baseline and again after the intervention.
2. Time to lactogenesis onset: This outcome was defined as the time from delivery to the mother’s perception of copious milk secretion or the onset of lactogenesis II, expressed in hours. It was used to evaluate the establishment of early postpartum lactation.
3. Early exclusive breastfeeding: Early exclusive breastfeeding outcomes included the exclusive breastfeeding rate at 24 h postpartum and the exclusive breastfeeding rate before hospital discharge. Exclusive breastfeeding was defined as feeding the newborn only breast milk, without supplementation with formula, water, or any other food.

#### Secondary outcomes

1. Social support level: Social support was assessed using the Maternity Social Support Scale (MSSS) [21]. Higher scores indicate higher perceived social support among pregnant and postpartum women. A total score of 0–18 was classified as low social support, 19–24 as moderate social support, and >24 as adequate social support. 
2. Breastfeeding behavior prediction: Breastfeeding behavior prediction was assessed using the Breastfeeding Attrition Prediction Tool (BAPT) [22]. Developed by Janke and based on the Theory of Planned Behavior, the BAPT includes the domains of social and professional support, positive attitudes, negative attitudes, and breastfeeding self-control. Scores for each domain were calculated by summing the corresponding item scores according to the original scoring method. Higher scores in the social and professional support, positive attitude, and breastfeeding self-control domains indicate more favorable breastfeeding-related psychosocial characteristics, whereas higher scores in the negative attitude domain indicate stronger negative attitudes toward breastfeeding. Therefore, BAPT domain scores were interpreted separately rather than combined into a single total score. It was used to evaluate participants’ propensity to engage in breastfeeding behavior and the related psychosocial factors.
3. Delayed lactogenesis: The incidence of delayed onset of lactogenesis II was recorded in both groups and compared between groups. Delayed lactogenesis was generally defined as the absence of obvious copious milk secretion by 72 h postpartum.

#### Postpartum maternal and neonatal outcomes and safety monitoring

To evaluate the safety of the AME intervention, maternal and neonatal outcomes were recorded and compared between the two groups, including gestational age at delivery, mode of delivery, use of labor analgesia, neonatal sex, birth weight, admission to the neonatal intensive care unit (NICU), and Apgar scores after birth. In addition, participants were closely monitored during the intervention for abnormalities such as sustained excessive uterine contractions, markedly decreased fetal movement, and vaginal bleeding. Safety monitoring included participant self-monitoring during home practice and daily review of practice logs by the research team. AME was discontinued when regular uterine contractions, persistent abdominal pain, vaginal bleeding, leakage of fluid, markedly decreased fetal movement, or other abnormal symptoms occurred, and obstetric assessment was arranged when clinically indicated. If any of these events occurred, the intervention was stopped immediately and appropriate management was provided.

#### Assessment time points

All participants completed the baseline assessment at enrollment. In the control group, post-intervention assessment was conducted after completion of routine prenatal health education. In the intervention group, post-intervention assessment was performed after participants had passed the in-clinic AME skills assessment and completed six consecutive practice sessions. Postpartum follow-up was conducted to collect data on feeding methods, time to lactogenesis onset, and maternal and neonatal outcomes in both groups. Safety-related information during home AME practice was prospectively collected from daily practice logs and participant reports. To ensure data consistency, all questionnaires were administered and collected by uniformly trained researchers using standardized instructions, and outcome data were verified against outpatient and inpatient medical records.

### Statistical analysis

Data were analyzed using SPSS version 26.0. Continuous variables were first tested for normality. Normally distributed data were expressed as mean ± standard deviation (SD); between-group comparisons were performed using the independent-samples t test, and within-group pre- and post-intervention comparisons were analyzed using the paired t test. Non-normally distributed data were expressed as median (interquartile range) and were compared between groups using the Mann–Whitney U test and within groups using the Wilcoxon signed-rank test. Categorical variables were presented as frequencies and percentages and were compared using the chi-square test or Fisher’s exact test, as appropriate. Ordinal data were analyzed using the rank-sum test. All statistical tests were two-sided, and a P value < 0.05 was considered statistically significant.

## Results

### Comparison of baseline characteristics between the two groups

**Fig. 1:**
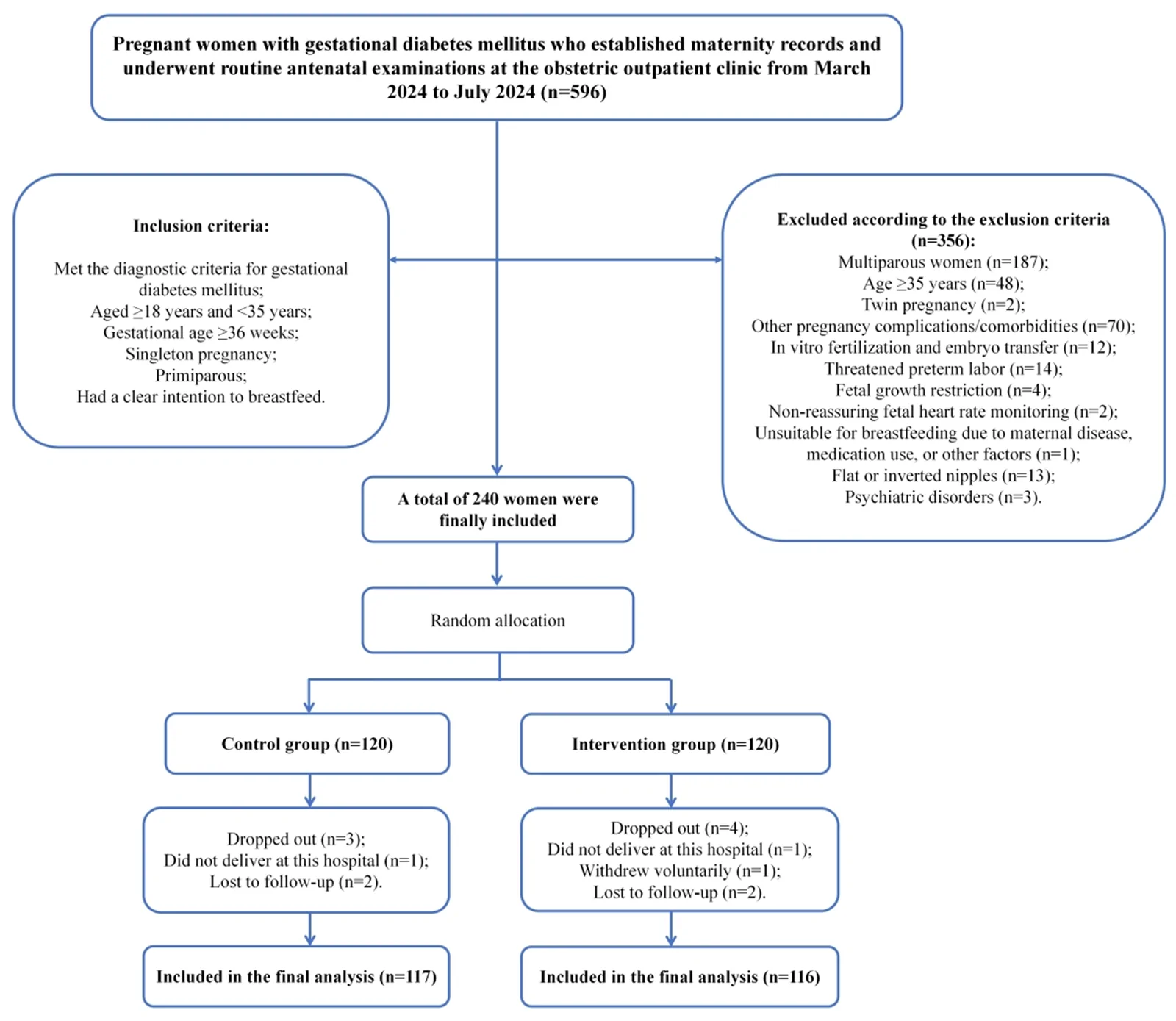
Flow chart of participant enrollment and follow-up

A total of 240 pregnant women with GDM were enrolled and randomly assigned to the intervention group or the control group at a 1:1 ratio. During the study period, 7 participants dropped out because of voluntary withdrawal, delivery outside the study hospital, or loss to follow-up. Ultimately, 233 participants completed the entire study protocol, yielding an overall attrition rate of 2.9%. Of these, 116 were in the intervention group and 117 in the control group (Fig. 1).

The mean age was 30.03 ± 2.51 years in the control group and 30.29 ± 2.45 years in the intervention group. The pre-pregnancy BMI was comparable between the control and intervention groups (21.38 ± 3.50 kg/m² [range, 15.82–31.46] vs. 21.28 ± 3.38 kg/m² [range, 15.64–30.92]), as was gestational weight gain (12.36 ± 4.10 vs. 12.04 ± 3.20 kg). Baseline glycemic profiles on the 75-g OGTT were also similar between groups, including fasting plasma glucose (4.92 ± 0.45 vs. 4.89 ± 0.43 mmol/L), 1-h plasma glucose (10.12 ± 1.05 vs. 10.08 ± 1.00 mmol/L), and 2-h plasma glucose (8.61 ± 0.83 vs. 8.55 ± 0.79 mmol/L). None of these differences were statistically significant (all *P* > 0.05). In both groups, most participants were of Han ethnicity, married, held a bachelor’s degree, and were company employees. Annual per-capita household income was most commonly between 150,000 and < 200,000 CNY. The proportions of women planning exclusive breastfeeding during hospitalization were 64.1% in the control group and 65.5% in the intervention group. In addition, the distribution of payment source and maternity leave duration was comparable between the two groups, with no statistically significant differences observed (all *P* > 0.05), indicating good baseline comparability (Table 1).

**Table 1:** Comparison of baseline characteristics between the two groups of pregnant women with GDM

Variable	Control group (*n* = 117)	Intervention group (*n* = 116)	Test statistic	*P* value
Age (years, mean ± SD)	30.03 ± 2.51	30.29 ± 2.45	t = 0.797	0.426
Pre-pregnancy BMI (kg/m², mean ± SD [range])	21.38 ± 3.50 [15.82–31.46]	21.28 ± 3.38 [15.64–30.92]	t=-0.231	0.818
Gestational weight gain (kg, mean ± SD)	12.36 ± 4.10	12.04 ± 3.20	t=-0.682	0.496
Fasting plasma glucose on OGTT (mmol/L, mean ± SD)	4.92 ± 0.45	4.89 ± 0.43	t=-0.520	0.603
1-h plasma glucose on OGTT (mmol/L, mean ± SD)	10.12 ± 1.05	10.08 ± 1.00	t=-0.298	0.766
2-h plasma glucose on OGTT (mmol/L, mean ± SD)	8.61 ± 0.83	8.55 ± 0.79	t=-0.566	0.572
**Ethnicity [n (%)]**				
Han ethnicity	114(97.4)	111(95.7)	χ²=0.138	0.71
Ethnic minority	3(2.6)	5(4.3)		
**Marital status [n (%)]**				
Unmarried	2(1.7)	4(3.4)	——	0.564
Married	114(97.4)	112(96.6)		
Divorced	1(0.9)	0(0.0)		
**Educational level [n (%)]**				
Junior high school or below	1(0.9)	1(0.9)	χ²=5.773	0.217
Senior high school / technical school / secondary vocational school	2(1.7)	1(0.9)		
Junior college	31(26.5)	17(14.7)		
Bachelor’s degree	60(51.3)	73(62.9)		
Master’s degree or above	23(19.7)	24(20.7)		
**Occupation [n (%)]**				
Civil servant	2(1.7)	4(3.4)	χ²=1.560	0.906
Healthcare worker	3(2.6)	3(2.6)		
Teacher	8(6.8)	11(9.5)		
Company employee	81(69.2)	79(68.1)		
Business owner	5(4.3)	4(3.4)		
Self-employed / freelancer	18(15.4)	15(12.9)		
**Annual per-capita household income [10**,**000 CNY/year**,** n (%)]**				
5 to < 10	2(1.7)	3(2.6)	χ²=7.068	0.07
10 to < 15	34(29.1)	28(24.1)		
15 to < 20	58(49.6)	45(38.8)		
≥ 20	23(19.7)	40(34.5)		
**Planned feeding method during hospitalization [n (%)]**				
Exclusive breastfeeding	75(64.1)	76(65.5)	χ²=0.051	0.821
Mixed feeding	42(35.9)	40(34.5)		
**Payment source [n (%)]**				
Urban medical insurance	113(96.6)	111(95.7)	χ²=0.215	0.898
Rural medical insurance	2(1.7)	3(2.6)		
Self-pay	2(1.7)	2(1.7)		
**Maternity leave duration [days**,** n (%)]**				
< 98	5(4.3)	5(4.3)	χ²=0.041	0.98
98 to 158	56(47.9)	54(46.6)		
> 158	56(47.9)	57(49.1)		

Note: Continuous variables were compared using the independent-samples t test. Categorical variables were compared using the chi-square test or Fisher’s exact test, as appropriate. “—” indicates that Fisher’s exact test was used. BMI, body mass index; GDM, gestational diabetes mellitus; OGTT, oral glucose tolerance test; SD, standard deviation

### Comparison of prenatal breastfeeding self-efficacy between the two groups

Before the intervention, the total prenatal breastfeeding self-efficacy scores were comparable between the two groups [control group: (54.47 ± 4.29) points; intervention group: (54.63 ± 4.68) points], and no statistically significant differences were observed in any of the four domains, namely behavioral perseverance, skills and needs, information acquisition, and embarrassment (all *P* > 0.05). After the intervention, the total score in the intervention group increased to (69.27 ± 7.30) points, which was significantly higher than that in the control group [(55.26 ± 4.42) points, *P* < 0.001]. Scores in the behavioral perseverance, skills and needs, information acquisition, and embarrassment domains also rose to (17.09 ± 3.49), (21.59 ± 2.19), (20.51 ± 2.02), and (10.09 ± 1.39) points, respectively, all of which were significantly higher than those in the control group (all *P* < 0.001). Within-group comparisons showed that only the information acquisition domain increased modestly in the control group (*P* = 0.039), whereas no significant changes were found in the other measures. In contrast, both the total score and all domain scores improved significantly in the intervention group (all *P* < 0.001) (Table 2).

**Table 2:** Comparison of prenatal breastfeeding self-efficacy scores between the two groups of pregnant women with GDM

Variable	Control group (*n* = 117)	Intervention group (*n* = 116)	Test statistic	Between-group *P* value
**Total score (points, mean ± SD)**				
Before intervention	54.47 ± 4.29	54.63 ± 4.68	t = 0.271	0.787
After intervention	55.26 ± 4.42	69.27 ± 7.30	t = 17.689	< 0.001
Within-group comparison	t = 1.580, *P* = 0.117	t = 18.958, *P* < 0.001		
**Behavioral perseverance domain (points**,** mean ± SD)**				
Before intervention	12.30 ± 2.33	12.52 ± 2.80	t = 0.647	0.518
After intervention	12.45 ± 2.30	17.09 ± 3.49	t = 11.973	< 0.001
Within-group comparison	t = 0.577, *P* = 0.565	t = 12.950, *P* < 0.001		
**Skills and needs domain (points**,** mean ± SD)**				
Before intervention	17.57 ± 2.21	17.34 ± 2.82	t = -0.713	0.476
After intervention	17.91 ± 1.84	21.59 ± 2.19	t = 13.894	< 0.001
Within-group comparison	t = 1.149, *P* = 0.253	t = 12.526, *P* < 0.001		
**Information acquisition domain (points**,** mean ± SD)**				
Before intervention	16.26 ± 2.11	16.17 ± 1.52	t = -0.349	0.728
After intervention	18.79 ± 1.90	20.51 ± 2.02	t = 14.517	< 0.001
Within-group comparison	t = 4.206, *P* = 0.039	t = 19.526, *P* < 0.001		
**Embarrassment domain (points**,** mean ± SD)**				
Before intervention	8.26 ± 1.88	8.60 ± 1.40	t = 1.603	0.11
After intervention	8.56 ± 1.28	10.09 ± 1.39	t = 8.747	< 0.001
Within-group comparison	t = 1.294, *P* = 0.198	t = 9.681, *P* < 0.001		

Note: Between-group comparisons were performed using the independent-samples t test, and within-group pre- and post-intervention comparisons were performed using the paired t test. SD, standard deviation

### Comparison of lactogenesis onset between the two groups

In the present study, the time to lactogenesis onset was (41.15 ± 21.94) h in the intervention group and (55.74 ± 23.29) h in the control group, indicating that lactogenesis onset occurred approximately 14.59 h earlier in the intervention group; the between-group difference was statistically significant (*P* < 0.05). The distribution of lactogenesis onset time also differed significantly between the two groups (χ²=26.486, *P* < 0.001). In the intervention group, lactogenesis onset was more frequently concentrated within 48 h postpartum, with 24.1% occurring within ≤ 24 h and 44.0% occurring between > 24 h and ≤ 48 h. In contrast, the control group showed a predominance of onset between > 48 h and < 72 h, accounting for 39.3%, and a higher proportion of onset at ≥ 72 h (24.8% vs. 8.6%). Further comparison of the incidence of delayed onset of lactogenesis II showed that 10 women (8.6%) in the intervention group experienced delayed lactogenesis II, which was significantly lower than the 29 women (24.8%) in the control group (χ²=10.922, *P* = 0.001). Correspondingly, the proportion without delayed onset of lactogenesis II was higher in the intervention group than in the control group (91.4% vs. 75.2%). These findings suggest that AME may promote earlier lactogenesis onset and reduce the risk of delayed onset of lactogenesis in women with GDM (Table 3).

**Table 3:** Comparison of lactogenesis onset between the two groups of women with GDM

Variable	Control group (*n* = 117)	Intervention group (*n* = 116)	Test statistic	*P* value
**Time to lactogenesis onset (h**,** mean ± SD)**	55.74 ± 23.29	41.15 ± 21.94	t = -4.922	< 0.001
**Distribution of time to lactogenesis onset [n (%)]**				
≤ 24 h	11(9.4)	28(24.1)	χ² = 26.486	< 0.001
> 24 h and ≤ 48 h	31(26.5)	51(44.0)		
> 48 h and < 72 h	46(39.3)	27(23.3)		
≥ 72 h	29(24.8)	10(8.6)		
**Delayed onset of lactogenesis II [n (%)]**				
No delayed onset of lactogenesis II	88(75.2)	106(91.4)	χ² = 10.922	0.001
Delayed onset of lactogenesis II	29(24.8)	10(8.6)		

Note: Time to lactogenesis onset was compared using the independent-samples t test. Categorical variables were compared using the chi-square test. SD, standard deviation

### Comparison of early postpartum feeding status between the two groups

The distribution of feeding patterns differed significantly between the two groups both at 24 h postpartum and before hospital discharge (χ²=7.121, *P* = 0.028; χ²=9.036, *P* = 0.011, respectively). At 24 h postpartum, the exclusive breastfeeding rate was 81.9% (95/116) in the intervention group, which was higher than the 66.7% (78/117) observed in the control group. Correspondingly, the proportion of artificial feeding decreased from 30.8% in the control group to 16.4% in the intervention group, whereas the proportion of mixed feeding was comparable between the two groups. Before maternal discharge, the intervention group still maintained a higher exclusive breastfeeding rate than the control group [43.1% (50/116) vs. 28.2% (33/117)], while the proportion of artificial feeding remained lower [9.5% (11/116) vs. 21.4% (25/117)]. Although the exclusive breastfeeding rate declined over the course of hospitalization in both groups, the intervention group consistently showed more favorable early in-hospital feeding outcomes, suggesting that the AME intervention may facilitate the establishment and maintenance of early exclusive breastfeeding in women with GDM (Table 4).

**Table 4:** Comparison of early postpartum feeding status between the two groups of women with GDM

Variable	Control group (*n* = 117)	Intervention group (*n* = 116)	Test statistic	*P* value
**Feeding status at 24 h postpartum [** ***n*** ** (%)]**				
Exclusive breastfeeding	78(66.7)	95(81.9)	χ² = 7.121	0.028
Mixed feeding	3(2.6)	2(1.7)		
Artificial feeding	36(30.8)	19(16.4)		
**Feeding status before maternal discharge [n (%)]**				
Exclusive breastfeeding	33(28.2)	50(43.1)	χ² = 9.036	0.011
Mixed feeding	59(50.4)	55(47.4)		
Artificial feeding	25(21.4)	11(9.5)		

Note: Between-group differences in feeding status were compared using the chi-square test

### Comparison of breastfeeding behavior prediction scores between the two groups

As shown in Table 5, before the intervention, there were no statistically significant differences between the two groups in the four domains of the Breastfeeding Attrition Prediction Tool, namely social and professional support, negative attitude, positive attitude, and breastfeeding self-control (all *P* > 0.05). After the intervention, scores in the social and professional support, positive attitude, and self-control domains were all significantly higher in the intervention group than in the control group, reaching (48.97 ± 3.53), (51.27 ± 4.64), and (37.14 ± 4.65) points, respectively (all *P* < 0.001). In contrast, the score for the negative attitude domain was significantly lower in the intervention group than in the control group [(49.70 ± 7.27) vs. (57.77 ± 4.01), *P* < 0.001]. Within-group comparisons showed no statistically significant pre- to post-intervention changes in any domain in the control group, whereas all four domains improved significantly in the intervention group (all *P* < 0.001). These findings suggest that AME may reduce negative expectations regarding breastfeeding while enhancing positive attitudes, perceived social support, and behavioral control, reflecting a more favorable breastfeeding-related psychosocial profile.

**Table 5:** Comparison of breastfeeding behavior prediction scores between the two groups of pregnant women with GDM

Variable	Control group (*n* = 117)	Intervention group (*n* = 116)	Test statistic	Between-group *P* value
**Social and professional support domain (points, mean ± SD)**				
Before intervention	43.16 ± 3.79	43.05 ± 4.76	t = -0.196	0.844
After intervention	43.94 ± 3.35	48.97 ± 3.53	t = 11.160	< 0.001
Within-group comparison	t = 1.616, *P* = 0.109	t = 10.840, *P* < 0.001		
**Negative breastfeeding attitude domain (points**,** mean ± SD)**				
Before intervention	58.67 ± 3.78	58.52 ± 6.01	t = -0.227	0.821
After intervention	57.77 ± 4.01	49.70 ± 7.27	t = -10.479	< 0.001
Within-group comparison	t = 1.878, *P* = 0.063	t = 9.644, *P* < 0.001		
**Positive breastfeeding attitude domain (points**,** mean ± SD)**				
Before intervention	47.76 ± 3.52	47.28 ± 3.85	t = -0.987	0.325
After intervention	48.47 ± 3.30	51.27 ± 4.64	t = 5.302	< 0.001
Within-group comparison	t = 1.568, *P* = 0.120	t = 7.141, *P* < 0.001		
**Breastfeeding self-control domain (points**,** mean ± SD)**				
Before intervention	28.10 ± 5.27	28.30 ± 6.61	t = 0.254	0.800
After intervention	28.91 ± 3.46	37.14 ± 4.65	t = 15.301	< 0.001
Within-group comparison	t = 1.471, *P* = 0.144	t = 12.146, *P* < 0.001		

Note: Between-group comparisons were performed using the independent-samples t test, and within-group pre- and post-intervention comparisons were performed using the paired t test. SD, standard deviation

### Comparison of postpartum maternal and neonatal outcomes and safety monitoring between the two groups

Overall, postpartum maternal and neonatal outcomes were comparable between the two groups. Gestational age at delivery was 39.55 ± 0.73 weeks in the intervention group and 39.53 ± 0.72 weeks in the control group, while birth weight was 3342.39 ± 335.43 g and 3302.59 ± 345.20 g, respectively; none of these differences reached statistical significance (all *P* > 0.05). The rate of vaginal delivery was slightly higher in the intervention group than in the control group (69.0% vs. 61.5%), although the difference was not statistically significant (*P* = 0.234). No significant between-group differences were observed in the use of labor analgesia, neonatal sex distribution, NICU admission rate, or 1-min and 5-min Apgar scores (all *P* > 0.05). During the study period, no cases of sustained excessive uterine contractions, markedly decreased fetal movement, or vaginal bleeding were observed in either group. Safety monitoring during the intervention period showed no observed cases of sustained excessive uterine contractions, markedly decreased fetal movement, vaginal bleeding, leakage of fluid, persistent abdominal pain, or emergency obstetric assessment due to AME-related safety concerns. No AME-related maternal or neonatal adverse events were identified during safety monitoring throughout the study period. These findings indicate that the AME intervention did not increase the risk of adverse maternal or neonatal outcomes and demonstrated short-term feasibility in this study population (Table 6).

**Table 6:** Comparison of maternal and neonatal outcomes between the two groups of women with GDM

Variable	Control group (*n* = 117)	Intervention group (*n* = 116)	Test statistic	*P* value
Gestational age at delivery (weeks, mean ± SD)	39.53 ± 0.72	39.55 ± 0.73	t = 0.170	0.865
Birth weight (g, mean ± SD)	3302.59 ± 345.20	3342.39 ± 335.43	t = 0.893	0.373
**Mode of delivery [n (%)]**				
Vaginal delivery	72(61.5)	80(69.0)	χ² = 1.417	0.234
Cesarean section	45(38.5)	36(31.0)		
**Labor analgesia [n (%)]**				
Yes	105(89.7)	105(90.5)	χ² = 0.039	0.843
No	12(10.3)	11(9.5)		
**Neonatal sex [n (%)]**				
Male	54(46.2)	64(55.2)	χ² = 1.895	0.169
Female	63(53.8)	52(44.8)		
Neonatal admission to the NICU [n (%)]	41(35.0)	39(33.6)	χ² = 0.052	0.819
1-min Apgar score [points, M (P25, P75)]	10.00(10.00, 10.00)	10.00(10.00, 10.00)	Z = -0.682	0.56
5-min Apgar score [points, M (P25, P75)]	10.00(10.00, 10.00)	10.00(10.00, 10.00)	Z = -1.004	0.315

Note: Continuous variables were compared using the independent-samples t test when normally distributed. Categorical variables were compared using the chi-square test. Non-normally distributed variables were compared using the Mann–Whitney U test. NICU, neonatal intensive care unit; SD, standard deviation; M, median; P25, 25th percentile; P75, 75th percentile

### Comparison of social support levels between the two groups

Before the intervention, no statistically significant differences were observed between the two groups in either the total social support score or the distribution of social support levels (both *P* > 0.05). After the intervention, the total social support score in the intervention group was significantly higher than that in the control group [(26.71 ± 1.74) vs. (23.97 ± 2.04), *P* < 0.001] and was also significantly increased compared with baseline (*P* < 0.001), whereas no significant change was found in the control group (*P* = 0.569). The distribution of social support levels showed a similar pattern: the proportion of participants with “adequate” social support in the intervention group increased from 39.7% to 88.8%, which was markedly higher than the 41.0% observed in the control group, with a statistically significant between-group difference (χ²=58.376, *P* < 0.001). These findings suggest that the AME intervention not only improved participants’ cognitive and behavioral preparedness but also enhanced their perceived support from family members, peers, and healthcare professionals (Table 7).

**Table 7:** Comparison of social support levels between the two groups of pregnant women with GDM

Variable	Control group (*n* = 117)	Intervention group (*n* = 116)	Test statistic	Between-group *P* value
**Total social support score (points, mean ± SD)**				
Before intervention	23.83 ± 1.78	23.96 ± 2.36	t = 0.467	0.641
After intervention	23.97 ± 2.04	26.71 ± 1.74	t = 10.969	< 0.001
Within-group comparison	t = 0.570, *P* = 0.569	t = 10.274, *P* < 0.001		
**Social support level [n (%)]**				
**Before intervention**				
Low	4(3.4)	3(2.6)	χ² = 0.436	0.804
Moderate	71(60.7)	67(57.8)		
Adequate	42(35.9)	46(39.7)		
**After intervention**				
Low	1(0.9)	0(0.0)	χ² = 58.376	< 0.001
Moderate	68(58.1)	13(11.2)		
Adequate	48(41.0)	103(88.8)		

Note: Total social support scores were compared using the independent-samples t test, and social support levels were compared using the chi-square test. Social support was categorized as low (0–18 points), moderate (19–24 points), or adequate (> 24 points). SD, standard deviation

## Discussion

Women with gestational diabetes mellitus (GDM) often face delayed lactogenesis onset, lower exclusive breastfeeding rates, and failure to achieve breastfeeding goals; therefore, targeted perinatal interventions are particularly important [10]. In recent years, antenatal milk expression (AME), as a lactation preparation strategy initiated in late pregnancy, has attracted increasing attention. However, previous studies have not been entirely consistent in their evaluation of its benefits in women with diabetic pregnancies. Randomized controlled trials have suggested that AME has a favorable safety profile [18], whereas subsequent follow-up studies have indicated that its effects on early exclusive breastfeeding, lactogenesis onset, and breastfeeding experience may still be influenced by factors such as the timing of the intervention, its intensity, and the support system provided [23]. Using a randomized controlled design, the present study demonstrated that AME not only improved prenatal breastfeeding self-efficacy and perceived social support in women with GDM, but also shortened the time to lactogenesis onset and increased the rate of early postpartum exclusive breastfeeding. These findings suggest that AME may enhance both prenatal feeding-related cognition and psychological readiness in women with GDM while also promoting the establishment of early postpartum breastfeeding, thereby providing clinical evidence to optimize perinatal breastfeeding support strategies for this population.

### Effect of AME on improving prenatal breastfeeding self-efficacy

Breastfeeding self-efficacy refers to a woman’s confidence in her ability to successfully carry out breastfeeding behaviors and is an important psychological determinant of breastfeeding initiation and duration. In women with GDM, it has particular clinical relevance for both the establishment and maintenance of breastfeeding [24]. In the present study, total PBSES scores as well as the domain scores for behavioral perseverance, skills and needs, information acquisition, and embarrassment all increased significantly after the intervention in the AME group and were consistently higher than those in the control group. These findings indicate that AME not only improved overall confidence in breastfeeding but also strengthened women’s perceived mastery of specific breastfeeding skills, information preparedness, and persistence in breastfeeding-related behaviors. This is consistent with the findings of You et al. [14], who reported that individualized breastfeeding education based on self-efficacy theory improved breastfeeding self-efficacy and exclusive breastfeeding outcomes in women with GDM. These results further support the potential role of AME in enhancing breastfeeding self-efficacy in this population. The observed improvement in self-efficacy may be attributable to repeated skills training and reinforcement through successful experience. This mechanism is consistent with social cognitive theory, whereby mastery experiences and observational learning enhance women’s confidence in breastfeeding and their willingness to persist [25, 26].

### AME promotes earlier lactogenesis onset and reduces the risk of delay

The onset of lactogenesis is a critical step in the establishment of breastfeeding, and any delay may adversely affect milk production and the subsequent duration of breastfeeding. This problem is particularly common among women with GDM [15, 27]. In the present study, women in the AME group experienced significantly earlier lactogenesis onset and a markedly lower incidence of delayed lactogenesis than those in the control group, supporting the beneficial role of AME in facilitating the early course of breastfeeding. Compared with the findings of Demirci et al. [28], the present study further suggests that AME may reduce reliance on early formula supplementation after birth and promote more timely lactogenesis onset in women with GDM. The mechanism by which AME advances lactogenesis onset is unlikely to be attributable to a single factor; rather, it may result from the combined effects of repeated breast stimulation, rehearsal of breastfeeding skills, and the earlier establishment of psychological adaptation [29]. This process may not only help mothers enter a breastfeeding state more quickly after delivery but also enhance their confidence in coping with early feeding difficulties, thereby further reducing the risk of delayed lactogenesis.

### AME improves the establishment and in-hospital maintenance of early exclusive breastfeeding

The exclusive breastfeeding rate is an important indicator for evaluating the effectiveness of breastfeeding interventions [30]. Women with GDM often face barriers such as delayed lactogenesis, psychological stress, and insufficient environmental support during breastfeeding, all of which contribute to lower exclusive breastfeeding rates [31]. In the present study, exclusive breastfeeding rates at both 24 h postpartum and before hospital discharge were significantly higher in the intervention group than in the control group, indicating the advantage of AME in promoting the establishment of early exclusive breastfeeding and its maintenance during hospitalization in women with GDM. These findings are consistent with those reported by Moorhead et al. [23], who showed that antenatal milk expression may improve both the exclusivity and continuation of early postpartum breastfeeding in women with gestational diabetes. Despite this between-group advantage, the exclusive breastfeeding rate declined from 24 h postpartum to before discharge in both groups, including the AME group. This pattern suggests that AME may facilitate early breastfeeding initiation and lactogenesis onset but may not be sufficient as a stand-alone strategy to maintain exclusive breastfeeding throughout the immediate postpartum hospitalization period. The decline may be related to maternal fatigue after delivery, perceived insufficient milk supply, difficulty with latch or positioning, neonatal feeding assessment and glycemic monitoring in infants born to women with GDM, and clinically indicated supplementation during hospitalization. Longer postpartum hospital stay may also increase opportunities for breastfeeding difficulties to be identified and for supplementation to be introduced when needed. Accordingly, the pre-discharge exclusive breastfeeding rate should be interpreted as an early in-hospital breastfeeding outcome rather than evidence of sustained exclusive breastfeeding. These findings support the role of AME as an antenatal preparatory intervention, while also indicating the need for continued bedside lactation support after delivery to maintain early breastfeeding gains.

### Improvement in behavioral prediction indicators suggests that AME influences the breastfeeding decision-making process

The initiation and maintenance of breastfeeding are shaped by multiple psychosocial factors, including behavioral attitudes, social support, and perceived behavioral control. Together, these variables influence pregnant women’s decision-making regarding infant feeding and form the theoretical basis for behavioral prediction [32, 33]. In the present study, following AME intervention, the intervention group showed significantly higher scores in the domains of positive attitude, social and professional support, and self-control, together with a lower score in the negative attitude domain, suggesting that the intervention effectively optimized the psychological factors relevant to breastfeeding-related decision-making. Previous studies have shown that women who hold more positive attitudes toward breastfeeding, receive stronger family and professional support, and possess greater behavioral control and self-efficacy are more likely to initiate and sustain breastfeeding [34, 35]. Our findings are consistent with these observations, indicating that the effects of AME extend beyond physiological breast preparation and may also operate through reshaping maternal attitudes toward breastfeeding, strengthening perceived external support, and enhancing the sense of self-mastery, thereby influencing key components of the breastfeeding decision-making process.

### Enhanced social support and the external environmental effects of AME

As an important external factor determining the continuation of breastfeeding, social support plays a critical role in both the initiation and maintenance of breastfeeding behavior among pregnant and postpartum women [36]. In the present study, both the total MSSS score and the social support level of women in the intervention group improved markedly after the intervention, highlighting the positive effect of AME on the external support environment. Consistent with the findings of Leng et al. [37], spousal knowledge, attitudes, and behavioral involvement are all associated with breastfeeding outcomes, underscoring the indispensable role of partners in breastfeeding-related decision-making. Copeland et al. [38] further reported that peer supporters can enhance the acceptability of interventions by providing mothers with experiential sharing and emotional encouragement.

The AME intervention incorporated support beyond professional guidance. Spouses and primary family caregivers, including mothers or mothers-in-law when they were expected to provide postpartum care, were encouraged to participate in AME-related education. Such family involvement may have enhanced emotional support, practice reminders, assistance with milk storage, and shared decision-making regarding breastfeeding during the transition from pregnancy to the early postpartum period. Peer communication facilitated by the research team may also have helped reduce embarrassment and uncertainty related to breast expression and breastfeeding preparation. From the perspective of the social ecological model, AME may therefore influence both individual preparedness and the surrounding support environment, thereby strengthening the pathway from breastfeeding intention to actual breastfeeding behavior [39]. However, the present analysis did not separately quantify the relative contributions of spouses, peers, mothers, or mothers-in-law to changes in social support. The observed improvement in social support should therefore be interpreted as the overall effect of the AME-based support model rather than the effect of any single support source.

### Safety findings support the clinical feasibility of AME

In clinical practice, safety has consistently been the primary concern regarding the promotion of antenatal milk expression (AME) among women with gestational diabetes mellitus (GDM) [16]. In the present study, systematic evaluation showed no adverse obstetric outcomes attributable to AME, such as abnormal uterine contractions or vaginal bleeding, suggesting an overall favorable safety profile. During home practice, safety monitoring showed no observed cases of sustained excessive uterine contractions, markedly decreased fetal movement, vaginal bleeding, leakage of fluid, persistent abdominal pain, or emergency obstetric assessment due to AME-related safety concerns. No AME-related maternal or neonatal adverse events were identified during safety monitoring throughout the study period. In conjunction with previous reports, ensuring safety depends on the strict exclusion of women with threatened preterm labor, premature rupture of membranes, or other obstetric complications, together with dynamic monitoring throughout the intervention period [23, 40]. These findings support the short-term clinical feasibility of AME when implemented in carefully selected women with GDM under standardized instruction and safety monitoring.

### Limitations and future directions

Several limitations of this study should be acknowledged. First, this was a single-center randomized controlled trial with a relatively homogeneous sample, and the participants were mainly singleton, primiparous women with GDM managed primarily through dietary intervention alone; therefore, the generalizability of the findings remains limited. Second, because of the nature of the intervention, blinding of participants and intervention providers was not feasible. Although data collectors and statistical analysts were blinded, the possibility of bias cannot be completely excluded. Third, outcome assessment was concentrated mainly on the early postpartum period and during hospitalization, and long-term breastfeeding continuation and infant outcomes were not evaluated. Although AME was associated with higher exclusive breastfeeding rates at 24 h postpartum and before discharge, the decline in exclusive breastfeeding between these two time points indicates that the present findings cannot establish sustained exclusive breastfeeding beyond the immediate in-hospital period. Finally, some measures were based on self-reported questionnaires and may therefore have been influenced by subjective factors. Maternal breastfeeding self-efficacy and social support were not reassessed during the postpartum period, which limited interpretation of the psychosocial mechanisms underlying the decline in exclusive breastfeeding before discharge. Length of postpartum hospital stay and the intensity of postpartum lactation support were also not incorporated into the present analysis, although these factors may influence the identification of breastfeeding difficulties and the use of supplementation during hospitalization. In addition, the relative contributions of spouses, peers, mothers, and mothers-in-law to social support were not separately quantified. Although no AME-related adverse maternal or neonatal events were identified during safety monitoring throughout the study period, the sample was restricted to carefully selected women with GDM who were mainly managed by dietary intervention alone. These methodological considerations should be taken into account when interpreting the magnitude and durability of the intervention effect. Future multicenter studies with longer follow-up, postpartum psychosocial assessment, standardized adverse-event reporting, and more detailed evaluation of family and peer support are warranted to further validate these findings.

## Conclusion

This study demonstrated that antenatal milk expression (AME) improved prenatal breastfeeding self-efficacy, social support levels, and breastfeeding behavior prediction indicators in women with gestational diabetes mellitus (GDM). AME was also associated with earlier postpartum lactogenesis onset, a lower incidence of delayed onset of lactogenesis II, and higher exclusive breastfeeding rates at 24 h postpartum and before hospital discharge. However, the decline in exclusive breastfeeding from 24 h postpartum to discharge indicates that these findings should be interpreted as early in-hospital breastfeeding outcomes rather than evidence of sustained exclusive breastfeeding beyond discharge. No AME-related maternal or neonatal adverse events were identified during safety monitoring throughout the study period, supporting the short-term feasibility of AME in eligible women with GDM. These findings suggest that, when implemented with standardized instruction and safety monitoring, AME may serve as a useful antenatal component of perinatal breastfeeding support for eligible women with GDM.

## Supplementary Information

Below is the link to the electronic supplementary material.


Supplementary Material 1



Supplementary Material 2


## Data Availability

The data used to support the findings of this study are available from the corresponding author upon request.
