# Supplementary material for: Effect of antenatal milk expression on prenatal breastfeeding self-efficacy, lactogenesis onset, and early exclusive breastfeeding in pregnant women with gestational diabetes mellitus: a randomized controlled trial
Source: Int Breastfeed J. 2026 Aug 6;21:81. doi: 10.1186/s13006-026-00879-w (PMC13531841; doi:10.1186/s13006-026-00879-w)
Supplement: Supplementary file 2 [file 13006_2026_879_MOESM2_ESM.doc]

**产前母乳表达对妊娠期糖尿病孕妇产前母乳喂养自我效能、泌乳启动及早期纯母乳喂养的影响：一项随机对照试验**

研究单位：杭州市妇产科医院

主要研究者： 蔡锋成，贾张越，吴映瑛，曾梦雨，王振奕杨婧苛，徐萌艳，章一娜

承担科室：护理部

联系电话： 13968124842

参与单位：浙江大学附属妇产科医院，杭州市妇产科医院

版本号：1.0

版本日期：2024年1月10日

**方 案 摘 要**

| 项目名称 | 产前母乳表达对妊娠期糖尿病孕妇产前母乳喂养自我效能、泌乳启动及早期纯母乳喂养的影响：一项随机对照试验 |
| --- | --- |
| 研究目的 | 探讨产前母乳表达（AME）对妊娠期糖尿病（GDM）孕妇产前母乳喂养自我效能、泌乳启动及早期纯母乳喂养的影响。 |
| 研究对象 | 妊娠晚期（≥36周）的单胎、初产、通过饮食干预管理的GDM孕妇。 |
| 病例数 | 共240例，干预组和对照组各120例。 |
| 纳入标准 | 1. 符合GDM诊断标准；2. 年龄≥18岁且<35岁；3. 孕周≥36周；4. 单胎妊娠；5. 初产妇；6. 有明确的母乳喂养意愿。 |
| 排除标准 | 1. 合并其他妊娠并发症或内外科合并症；2. 先兆早产；3. 胎儿生长受限；4. 胎心监护异常；5. 因母体疾病或用药不宜母乳喂养；6. 扁平或凹陷乳头；7. 体外受精-胚胎移植受孕；8. 精神或心理疾病；9. 胎儿畸形。 |
| 试验设计 | 单中心、随机、开放、对照临床研究。对照组接受GDM常规产前护理及产后常规护理。干预组在对照组基础上，从孕36周后接受系统化的AME指导（包括健康宣教、技能示范与练习），每日两次（晨起和睡前），直至分娩。研究包含基线评估、干预后评估和产后随访。 |
| 评价指标 | 主要疗效评价指标：1. 产前母乳喂养自我效能（PBSES量表）； 2. 泌乳启动时间（小时）； 3. 早期纯母乳喂养率（产后24小时和出院前）。  次要疗效评价指标：1. 社会支持水平（MSSS量表）； 2. 母乳喂养行为预测指标（BAPT量表）； 3. 泌乳启动延迟（Ⅱ期）发生率。  安全性评价指标：产妇及新生儿结局（分娩孕周、分娩方式、出生体重、NICU入住率、Apgar评分等）及干预相关不良事件。 |
| 统计分析 | 使用SPSS 26.0软件。连续变量正态分布采用均数±标准差描述，组间比较使用独立样本t检验，组内前后比较使用配对t检验；非正态分布使用中位数（四分位间距）描述，组间比较使用Mann-Whitney U检验。分类变量使用频数和百分比描述，组间比较使用卡方检验或Fisher精确检验。所有检验为双侧，P<0.05为差异有统计学意义。 |
| 研究期限 | March and July 2024 |

**研究方案名称**

一、研究背景

妊娠期糖尿病（GDM）是孕期最常见的并发症之一，其患病率在全球范围内持续上升，在中国及其他中低收入国家亦呈显著增长趋势。GDM不仅增加不良围产期结局的风险，而且GDM孕妇更易出现泌乳启动延迟、纯母乳喂养率低、较早引入配方奶等问题，影响新生儿营养和母体母乳喂养信心。现有干预措施多聚焦于产后阶段，可能不足以提供充分的产前技能准备和信心建立。

产前母乳表达（AME）作为一项在妊娠晚期启动的泌乳准备策略，近年来被引入高危孕妇的护理中。既往研究表明AME安全可行，可能促进乳汁分泌、提高母乳喂养自我效能及产妇满意度，并有助于提高纯母乳喂养率。然而，关于AME是否能够改善中国内地GDM孕妇从产前到产后的整合性母乳喂养路径，尤其是产前母乳喂养自我效能、泌乳启动和早期纯母乳喂养的证据仍然有限。因此，本研究聚焦于AME对GDM孕妇上述结局的影响，以期为改善GDM孕妇的母乳喂养管理策略提供循证依据。

1. 研究目的

探讨产前母乳表达（AME）对妊娠期糖尿病（GDM）孕妇产前母乳喂养自我效能、泌乳启动及早期纯母乳喂养的影响。

1. 试验类型

本研究为单中心、随机、开放、对照（parallel-group）临床研究。

1. 研究对象及病例选择
1、纳入标准

（1）符合GDM诊断标准；（2）年龄≥18岁且<35岁；（3）孕周≥36周；（4）单胎妊娠；（5）初产妇；（6）有明确的母乳喂养意愿。

2、排除标准

（1）合并其他妊娠并发症或内外科合并症（如妊娠期高血压、甲状腺功能减退合并妊娠、前置胎盘、产前出血等）；（2）先兆早产；（3）胎儿生长受限；（4）胎心监护异常；（5）因母体疾病、用药或其他相关因素不宜母乳喂养；（6）扁平或凹陷乳头；（7）体外受精-胚胎移植受孕；（8）精神或心理疾病；（9）胎儿畸形。

1. 试验设计

干预方案：

对照组： 接受GDM常规产前护理（包括标准产前检查、饮食运动管理、血糖监测指导及母乳喂养相关健康教育）和产后常规护理。

干预组： 在对照组基础上，增加产前母乳表达（AME）干预。

培训阶段（孕≥36周）： 由经过统一培训的产科护士在门诊教育室进行一对一的AME指导，内容包括健康宣教、技能示范、监督练习和回示。培训时长约30-40分钟。邀请配偶及主要家庭照顾者参与教育。

家庭练习阶段（孕37周至分娩）： 包括乳房按摩（避开乳晕和乳头，每侧3-5分钟）和手挤奶（C字形，每侧5分钟，两侧交替）。每日两次（晨起及睡前）。参与者需记录每日练习日志，由研究团队每日在线审阅。

实施周期： 从孕36周后开始培训，孕37周起每日练习直至分娩。

随机化分组方法： 使用随机数字表法，由一名不参与招募、干预、评估和数据分析的研究者生成分配序列。采用按顺序编号、密封、不透明的信封进行分配隐藏。参与者按入组顺序分配研究编号，并依次开启信封确定分组。

1. 评价指标及评估方法

主要疗效评价指标：产前母乳喂养自我效能： 采用产前母乳喂养自我效能量表（PBSES）评估，含20个条目，总分20-100分，分数越高表明自我效能越高。泌乳启动时间： 定义为从分娩至母亲感知到乳汁大量分泌（即泌乳Ⅱ期启动）的时间，以小时为单位。早期纯母乳喂养率： 评估产后24小时和出院前的纯母乳喂养率。次要疗效评价指标：社会支持水平： 采用产妇社会支持量表（MSSS）评估。母乳喂养行为预测指标： 采用母乳喂养减员预测工具（BAPT）评估。泌乳启动延迟（Ⅱ期）发生率： 定义为产后72小时仍未出现明确的乳汁大量分泌。**安全性评价指标：**
产妇及新生儿结局（分娩孕周、分娩方式、产时镇痛使用、新生儿性别、出生体重、NICU入住率、Apgar评分）及干预相关不良事件（如持续过强宫缩、胎动明显减少、阴道出血等）。

**探索性指标：** 无。

1. 病例数

样本量基于两个独立比例比较的公式进行计算，以出院前纯母乳喂养率作为样本量估算的主要参数。参考既往研究中GDM产妇的母乳喂养结局及AME的预期效应量，设定双侧α=0.05，检验效能1-β=0.90，估算每组需96例。考虑约20%的预期脱落率，最终目标样本量设定为240例，每组120例。

1. 统计分析

对于连续型变量，将描述其例数、均值、标准差、中位数、第25及75百分位数等。对于离散型变量，将描述各类的例数及百分比。

根据分析指标的分布，如资料符合正态分布，采用参数检验（如独立样本t检验、配对t检验）；如资料不符合正态分布，采用非参检验（如Mann-Whitney U检验、Wilcoxon符号秩检验）。计量资料组内前后比较采用配对t检验或Wilcoxon符号秩检验，组间比较采用独立样本t检验或Mann-Whitney U检验；计数资料组间比较用卡方检验，必要时用Fisher精确概率法。除非另有说明，所有的统计检验都将使用α=0.05的双侧检验，P<0.05被认为差异有统计学意义。

1. 研究风险及处置预案

AME干预的潜在风险包括可能诱发宫缩、胎动异常、阴道出血等。为此，研究将严格执行排除标准（排除先兆早产、前置胎盘等高危因素）。干预期间，参与者需进行自我监测，研究团队每日在线审阅练习日志。若出现规律或持续性宫缩、胎动明显减少、阴道出血、流液、持续性腹痛或其他异常症状，应立即停止AME并寻求产科评估。既往研究提示，在严格筛选的低风险糖尿病孕妇中，AME的安全性良好。

十一、研究终止标准

1、出现任何研究者认为继续参与研究不符合研究参与者最佳利益的临床事件、实验室检查异常或并发疾病。
2、研究参与者出现规律宫缩、持续性腹痛、阴道出血、流液、胎动明显减少或其他需要紧急产科处理的症状。
3、研究者严重不遵从研究方案。
4、伦理委员会要求终止研究。

十二、不良事件及处置

**严重不良事件（Serious Adverse Event，SAE）**：指研究参与者接受试验用药品后出现死亡、危及生命、永久或者严重的残疾或者功能丧失、研究参与者需要住院治疗或者延长住院时间，以及先天性异常或者出生缺陷等不良医学事件。

一旦发生SAE，研究者应在发现后**24h内上报**并同时递交SAE报告表至临床研究伦理委员会审查不良事件，并记录在案；发生死亡病例应按医院规定及时向医务部门报告。

处理措施：研究者需**立即采取措施**，保证研究参与者及时接受临床治疗，并对其SAE的转归进行**跟踪随访直至痊愈或恢复至基线期水平**。

十三、药品编码及揭盲

本研究为开放标签试验，不涉及试验用药品编码及揭盲。

十四、质量控制

研究者将采用标准操作规程，以保证临床研究的质量控制和质量保证系统的实施。临床研究中所有观察结果和发现都将加以核实，以保证数据的可靠性，确保临床研究中各项结论来源于原始数据。在数据处理的每一阶段均采用质量控制，以保证所有数据可靠，处理正确。所有问卷由经过统一培训的研究人员使用标准化指导语进行发放和收集。结局数据将与门诊和住院病历进行核对验证。

十五、伦理学要求

1.临床研究开始前，试验方案需经伦理委员会审查，审查结果为同意，并签署批件后方可实施。

2.在试验期间，遵循WMA《赫尔辛基宣言》（2013）、CIOMS《人体生物医学研究国际伦理指南》（2016）和国家卫生计生委《涉及人的生命科学和医学研究伦理审查办法》（2023）。

3.在试验期间，临床研究方案、知情同意书、招募材料等的任何修改，均须得到伦理委员会审查同意后方可实施。

4.在每一位研究参与者入选本研究前，研究者必须向研究参与者详细介绍**现有的治疗方法**，本研究目的，试验过程与期限、检查操作、研究参与者预期可能的受益与风险，可能花费的金钱与时间、研究参与者被分配到不同的组别等。此外，研究者需要告知研究参与者参与此项试验完全是自愿原则，而且有权在试验的任何阶段随时退出试验而不会遭到歧视与报复，其医疗待遇与权益不会受到影响。

5.研究者经充分和详细解释试验的情况，由研究参与者或其法定代理人（对无行为能力的研究参与者）在知情同意书上签字并注明期，执行知情同意过程的研究者也需在知情同意书上签署姓名和日期，知情同意书一式两份，分别由研究参与者和研究者保存。

十八、参考文献

[1].	Buchanan, T.A., et al., What is Gestational Diabetes – Really? Diabetes, 2025. 74(7): p. 1037-1046.

[2].	Retnakaran, R., The Insulin-Like Growth Factor Axis: A New Player in Gestational Diabetes Mellitus? Diabetes, 2016. 65(11): p. 3246-3248.

[3].	McIntyre, H.D., et al., Gestational diabetes mellitus. Nature Reviews Disease Primers, 2019. 5(1): p. 47.

[4].	Modzelewski, R., et al., Gestational Diabetes Mellitus—Recent Literature Review. Journal of Clinical Medicine, 2022. 11(19): p. 5736.

[5].	Lin, J., et al., Trends in the Incidence of Gestational Diabetes Mellitus Among the Medicaid Population Before and During the COVID-19 Pandemic. Journal of Women's Health, 2024. 33(9): p. 1276-1282.

[6].	Chen, L., et al., Gestational Diabetes Mellitus: Its Epidemiology and Implication beyond Pregnancy. Current Epidemiology Reports, 2016. 3(1): p. 1-11.

[7].	Wu, X., H. Tiemeier and T. Xu, Trends in gestational diabetes prevalence in China from 1990 to 2024: a systematic review and meta-analysis. Reviews in Endocrine and Metabolic Disorders, 2025. 26(6): p. 1009-1021.

[8].	Wang, H., et al., IDF Diabetes Atlas: Estimation of Global and Regional Gestational Diabetes Mellitus Prevalence for 2021 by International Association of Diabetes in Pregnancy Study Group’s Criteria. Diabetes Research and Clinical Practice, 2022. 183: p. 109050.

[9].	Sweeting, A., et al., Epidemiology and management of gestational diabetes. The Lancet, 2024. 404(10448): p. 175-192.

[10].	Doughty, K.N. and S.N. Taylor, Barriers and benefits to breastfeeding with gestational diabetes. Seminars in Perinatology, 2020. 45(2): p. 151385.

[11].	Otter, G., et al., Promoting breastfeeding in women with gestational diabetes mellitus in high-income settings: an integrative review. International Breastfeeding Journal, 2024. 19(1): p. 4.

[12].	Qian, P., et al., Decision-making process of breastfeeding behavior in mothers with gestational diabetes mellitus based on health belief model. BMC Pregnancy and Childbirth, 2023. 23(1): p. 242.

[13].	Geddes, D.T., Z. Gridneva and S.L. Perrella, Breastfeeding after gestational diabetes mellitus: maternal, milk and infant outcomes. Current Opinion in Clinical Nutrition and Metabolic Care, 2025. 28(3): p. 257-262.

[14].	You, H., et al., Effects of breastfeeding education based on the self-efficacy theory on women with gestational diabetes mellitus. Medicine, 2020. 99(16): p. e19643.

[15].	Peng, Y., K. Zhuang and Y. Huang, Incidence and factors influencing delayed onset of lactation: a systematic review and meta-analysis. International Breastfeeding Journal, 2024. 19(1): p. 59.

[16].	Johnsen, M., et al., Antenatal breastmilk expression for women with diabetes in pregnancy - a feasibility study. International Breastfeeding Journal, 2021. 16(1): p. 56.

[17].	Wu, Q., X. Wang and F. Zhu, Effect of Milking Interventions at Different Stages on Breastfeeding Rate and Quality in Women with Gestational Diabetes Mellitus. International Journal of Women's Health, 2025: p. 23-2535.

[18].	Forster, D.A., et al., Advising women with diabetes in pregnancy to express breastmilk in late pregnancy (Diabetes and Antenatal Milk Expressing [DAME]): a multicentre, unblinded, randomised controlled trial. The Lancet, 2017. 389(10085): p. 2204-2213.

[19].	Of, I., et al., International Association of Diabetes and Pregnancy Study Groups Recommendations on the diagnosis and classification of hiperglycemia in pregnancy International Association of Diabetes and Pregnancy Study Groups Consensus Panel (IADPSG) Diabetes Care 2010 33 3 676 82 10.2337/dc09-1848. Diabetes care, 2010. 33: p. 676-682.

[20].	Wells, K.J., N.J. Thompson and A.S. Kloeblen-Tarver, Development and Psychometric Testing of the Prenatal Breast-feeding Self-efficacy Scale. Am J Health Behav, 2006. 30(2): p. 177-187.

[21].	Webster, J., et al., Measuring Social Support in Pregnancy: Can It Be Simple and Meaningful? Birth, 2000. 27(2): p. 97-101.

[22].	Janke, J.R., Development of the Breast‐Feeding Attrition Prediction Tool. NURSING RESEARCH, 1994. 43: p. 100–104.

[23].	Moorhead, A.M., et al., Breastfeeding outcomes at 3 months for women with diabetes in pregnancy: Findings from the Diabetes and Antenatal Milk Expressing randomized controlled trial. Birth: Issues in Perinatal Care, 2024. 51(3): p. 508-520.

[24].	McGovern, L.M., et al., An exploration of prenatal breastfeeding self-efficacy: a scoping review. International Journal of Behavioral Nutrition and Physical Activity, 2024. 21(1): p. 95.

[25].	Mohamad Pilus, F., et al., Effect of Face-to-Face and WhatsApp Communication of a Theory-Based Health Education Intervention on Breastfeeding Self-Efficacy (SeBF Intervention): Cluster Randomized Controlled Field Trial. JMIR mHealth and uHealth, 2022. 10(9): p. e31996.

[26].	Bai, Y.K., S. Lee and K. Overgaard, Critical Review of Theory Use in Breastfeeding Interventions. Journal of Human Lactation, 2019. 35(3): p. 478-500.

[27].	Halawa, M.R., et al., Impact of Diabetes in Pregnancy on Stage II Lactogenesis among Postpartum Diabetic Women Compared to Non-Diabetics. QJM: An International Journal of Medicine, 2024. 117(Supplement_1): p. hcae070.271.

[28].	Demirci, J.R., et al., Structured antenatal milk expression education for nulliparous pregnant people: results of a pilot, randomized controlled trial in the United States. International Breastfeeding Journal, 2022. 17(1): p. 50.

[29].	Hannan, F.M., et al., Hormonal regulation of mammary gland development and lactation. Nature Reviews Endocrinology, 2022. 19(1): p. 46-61.

[30].	Nixarlidou, E., et al., Clinical significance and main parameters promoting the breast‑feeding strategy (Review). Medicine international, 2024. 4(2): p. 14.

[31].	McGuinness, D., U. Daniel and D.O. Brien, Facilitators and barriers to establishing lactation among women with diabetes? Diabetic Medicine, 2025. 42(8): p. e70045.

[32].	Niroula, K. and E.M. McKinley, Approaching Breastfeeding Behavior Predictors Through Theory of Planned Behavior: Instrumentation and Validation of New Survey. The Journal of Perinatal & Neonatal Nursing, 2026. 40(1): p. 31-37.

[33].	Wu, L., et al., Understanding the psychosocial dilemma in breastfeeding: a narrative review of extended theory of planned behavior and its intervention strategies. Reproductive Health, 2025. 22(1): p. 46.

[34].	Rafizadeh, R., et al., Factors affecting breast-feeding practice among a sample of Iranian women: a structural equation modeling approach. Italian Journal of Pediatrics, 2019. 45(1): p. 147.

[35].	Sanchez, L.M., et al., Social media intervention for promoting breastfeeding among WIC participants. Food Science & Nutrition, 2023. 11(11): p. 6945-6954.

[36].	Page, A.E., E.H. Emmott and S. Myers, Testing the buffering hypothesis: Breastfeeding problems, cessation, and social support in the UK. American Journal of Human Biology, 2021. 34(2): p. e23621.

[37].	Leng, R.N.W., et al., Fathers’ Involvement in Their Wives’/Partners’ Breastfeeding: A Descriptive Correlational Study. Journal of Human Lactation, 2019. 35(4): p. 801-812.

[38].	Copeland, L., et al., Feasibility and acceptability of a motivational interviewing breastfeeding peer support intervention. Maternal & Child Nutrition, 2018. 15(2): p. e12703.

[39].	Costenbader, E., et al., ‘Breastfeeding Is Not the Sole Responsibility of Women’: A Qualitative Examination of the Supportive Environment for Breastfeeding in Nigeria Across Levels of the Socioecological Model. Maternal & Child Nutrition, 2025. 21(4): p. e70050.

[40].	Alyousefi, N., et al., Predictors of Prenatal Breastfeeding Self-Efficacy in Expectant Mothers with Gestational Diabetes Mellitus. International Journal of Environmental Research and Public Health, 2022. 19(7): p. 4115.
